# Supplementary material for: Abdominal obesity and association with sociodemographic, behavioral and clinical data in climacteric women assisted in primary care
Source: PLoS One. 2020 Aug 13;15(8):e0237336. doi: 10.1371/journal.pone.0237336 (PMC7425907; doi:10.1371/journal.pone.0237336)
Supplement: Attachment — Submitted filename: Response to Reviewers 2.docx [file pone.0237336.s002.docx]

Montes Claros, 07 de june de 2020.

Dear Editor and Reviewers.

Attending the reviewers’ recommendations, we described below the modifications made in the manuscript.

1) 1) Thank you for your resubmission. We note that the following request has not been addressed: We note that your recently published article (Marques, Maria Suzana, et al. "Health conditions associated with overweight in climacteric women." PloS one 14.12 (2019).), is related to the present study, as the same sample population was considered, and the same scale adopted. To meet PLOS ONE criteria on related manuscripts (http://journals.plos.org/plosone/s/submission-guidelines#loc-related-manuscripts), we would ask that the previous analysis is adequately mentioned and cited (not only in the Cover letter) in the present submission, and the rationale of these separate analyses is clearly discussed.

RESPONSE: We have added this information in the text. The text has been revised accordingly so that this information is described in more detail (line 88-99).

Sincerely,

The authors
