# Supplementary material for: Abdominal obesity and association with sociodemographic, behavioral and clinical data in climacteric women assisted in primary care
Source: PLoS One. 2020 Aug 13;15(8):e0237336. doi: 10.1371/journal.pone.0237336 (PMC7425907; doi:10.1371/journal.pone.0237336)
Supplement: Attachment — Submitted filename: Response to Reviewers 3.docx [file pone.0237336.s003.docx]

Montes Claros, 22 de july de 2020.

Dear Editor,

Attending the specific academic editor comments, we described below the modifications made in the manuscript.

1) Although the majority of comments were addressed appropriately, I think it would be a good idea to better state somewhere within lines 88-99 what is novel about this study by including a directional hypothesis statement (to match one included in the abstract and introduction), as this seems to be a re-analysis of data collected from an already-published cohort.

RESPONSE: We have added this information in the text. The text has been revised accordingly so that this information is described in more detail (line 94-98). When reviewing the text, some information was moved from paragraph to paragraph as noted in the lines (119-120) and (129-130). All changes are highlighted in red in the body of the text.

Sincerely,

The authors
